# “Everything is Tuberculosis” – Except the Law?

**DOI:** 10.1017/jme.2025.10128

**Published:** 2025

**Authors:** James G. Hodge

**Affiliations:** College of Law, Arizona State University, United States

**Keywords:** tuberculosis (TB), treatment, law, rights, prevention

## Abstract

In “Everything is Tuberculosis,” author John Green assesses the intricacies of the communicable condition, TB, as a source of significant morbidity and mortality globally over centuries. Despite available vaccines, treatments, and protocols, tens of millions are infected and over a million persons will die from TB in 2025 alone. In searching for answers to mitigate this global scourge, however, Green looks past a key factor — specifically the role of law — as a primary tool for prevention and control.

In his new book, “Everything is Tuberculosis: The History and Persistence of Our Deadliest Infection,”^1^ noted young-adult fiction writer John Green tackles a critical topic in a new literary genre with renewed hope for the future of millions of persons infected with tuberculosis (TB). Green’s objective, clarified at the very end of his text, is nothing less than to chart a course for public and private sectors to “work together to end [TB] and all other diseases of injustice.”^2^ It is a lofty goal that has evaded societies for millennia, but noble regardless.

Green’s treatment of the subject matter in his easy-to-read style is capturing readers of all ages internationally. His elucidation of the historical aspects of TB as a blight on global health over centuries is fascinating, even if it is well-documented elsewhere (as the author acknowledges).^3^ He neatly recites statistics on the morbidity and mortality of TB over the ages, brings current stories of TB survivors — and those less fortunate — to light, and proffers a profound case for greater focus on public health, research, and health care solutions to abate the continued global spread of the disease.

For a condition that is eminently treatable through existing drug regimens (in many cases), Green’s call to curb TB impacts is a social and moral imperative. In her review of the text, Rebecca Robins for *The New York Times* remarks on its timeliness given the 2025 deconstruction of the US Agency for International Development (USAID),^4^ not to mention President Trump’s intention to withdraw the US (and its vast resources) from the World Health Organization (WHO).^5^ These and other shifts in US policies carry significant global health repercussions, including escalating TB deaths that already exceed one million per annum.

Yet, for an author claiming “everything is TB,” it is remarkable just how little the text actually devotes to a core tool and foundation of TB prevention and control, specifically the role of law. As discussed below, Green fixates on science, medicine, and social forces as pivotal to effective TB abatement. No one doubts these conclusions. Matching available, efficacious medicines to patients in dire need of treatment makes sense. What Green does not seem to appreciate is how TB laws and policies are intricately interwoven into historic and modern approaches to curtailing this threat. Collaborating to “cure” the global spread of TB is not just about finding more funding or increasing access to medicines and doctors, but also about charting legal routes to assure the same.

## Focus on TB Prevention and Treatment

There is much to like in Green’s account of the interfusion of TB into all facets of people’s lives. As an infectious condition, TB has contributed to fear and panic among patients and their families, resulted in discrimination by others, and generated purported medical and public health strategies to combat the disease grounded in misperceptions of its causes, spread, and treatment. Like other infectious diseases, notably HIV/AIDS (as the author observes), TB knows no boundaries, impacts the poor and rich alike, and is a source for widespread stigmatization.

What’s exceptional about TB, however, is Green’s descriptions of positives about the condition itself. Known historically as “consumption,” TB is a wasting disease, robbing persons of their appetite and weight along the way. Consequently, some have depicted their infections as beneficent or “flattering.”^6^ Green notes how TB was viewed favorably by some for revealing one’s radiant beauty, particularly among women exhibiting a blanched skin tone, rosy cheeks, ruby lips, and wide eyes.^7^ While most felt cursed by TB, the poet John Keats, writer Charlotte Bronte, and others were deemed almost blessed by their infection.^8^ Green’s insights reveal the complexities of preventing and treating a disease both reviled and revered.

Green leaves no doubt, however, that TB is a scourge whose public health impacts exceed other infectious conditions globally. He personalizes TB’s true effects on the human condition through a series of accounts of patients and others he has met, interviewed, and befriended. The author spends considerable energy on extensive efforts undertaken over centuries to detect, prevent, and treat the disease. Medical and public health pioneers at the forefront of substantial achievements toward generating cures are celebrated in the text — and rightfully so.

## Promise and Pitfalls of Public Health Laws

Lost amid Green’s account, however, is the role law plays in historic and modern perceptions of TB. Public health officials uncover new epidemiological findings. Scientists and companies develop new drugs and vaccines. Doctors and nurses utilize new treatments. Philanthropists fund access to these treatments and providers. Yet each of these actors and TB patients themselves rely on effective public health laws and policies to effectuate these specific ends. Among other objectives, public health laws:

- authorize the collection of surveillance data that illuminate the scope of TB prevalence;
- set forth testing, screening, and social distancing measures to help limit its spread;
- protect the privacy of patient data to avert stigmatization;
- prohibit unwarranted discrimination against persons with TB (and other disabilities);^9^
- allow for adaptations in medical standards of care to find new treatments (including directly-observed therapies noted by Green);^10^
- buttress the scientific discovery of treatments with appropriate patient protections;
- approve the introduction and market use of vaccines, drugs and other preventatives or treatments;
- provide the infrastructure that promotes access to and use of safe and effective control measures; and
- require specific actors to intervene in the interests of patients and populations.^11^

Of course, laws also have supported inappropriate responses to TB over time, including interventions directly harming patients and their families (e.g., forcible treatments, unwarranted quarantine or isolation in sanatoriums, travel limitations, and adverse discrimination). Like medical experimentation surrounding TB patients, as Green observes, public health law has a checkered past. TB-specific laws and other general health measures have been used to deny patients’ rights, inhibit their movement, and target specific populations for special controls despite insufficient evidence of their risk to others.

Laws underlying medical treatment of infectious persons present particularly thorny issues.^12^ To the extent that TB is infectious among some patients, they need to be treated to safely participate in societal activities. Yet, treatments can take months, entail some risks to patients, and tend to require completion to be effective. TB treatment regimens are not for all, and not everyone who needs medical care will participate fully. In the United States, Supreme Court jurisprudence rejects government imposition of non-consensual, forced treatment^13^ (except in limited cases involving minors, wards, or prisoners).^14^ Tensions between protecting the public’s health and respecting personal autonomy escalate further among patients infected with multi-drug resistant (MDR) or extreme-drug resistant (XDR) strains.

Over years, multiple states have crafted legal compromises. While infectious TB patients may not be physically forced to undergo treatment, they can be isolated or detained (sometimes in jails) in rare cases where they refuse all treatment outright or fail to fully complete treatment regimens.^15^ Courts typically uphold isolation or detention of recalcitrant persons under state or local public health powers so long as procedural safeguards are met (e.g., notice, hearing, right to counsel or appeal).^16^ In an exceptional case in 1980, the West Virginia Supreme Court found that public health authorities improperly detained an infectious TB patient without adequate due process.^17^

A widely-publicized incident in 2007 involving a TB patient who evaded local, state, and federal efforts to limit his international travel led to significant US legal reforms. Andrew Speaker, an attorney living in Atlanta, home to the federal Centers for Disease Control and Prevention (CDC), avoided public health limitations to travel in Europe despite being suspected of harboring XDR-TB. Repeated attempts to seek his acquiescence with foreign and domestic health authorities eventually resulted in Speaker’s return to the US through Canada, at which point he was detained and successfully treated over weeks.^18^ His subsequent lawsuit against CDC alleging privacy and other violations was dismissed in 2012.^19^

The media storm surrounding Speaker’s case contributed to major legal reforms of CDC’s antiquated disease control regulations. After years of deliberations, CDC implemented new rules to clarify and enhance its powers in January 2017, just prior to the first inauguration of President Trump.^20^ Its regulations center on US airports and other transit locations and allow active screening techniques (e.g., observation, questioning, and review of suspect travelers’ documents and health records) coupled with enhanced social distancing powers (e.g., temporary apprehension, quarantine, and isolation).^21^ Though controversial these federal powers and their state equivalents have since been used to ascertain and address persons with TB and other infectious diseases.

## Law as Quintessential to TB Prevention and Control

Like modern advancements in science, medicine, and social norms, law is quintessential to ameliorating global morbidity and mortality tied to TB. Ongoing efforts through WHO, United Nations, and other global health entities to address TB prevalence require legal authorities including transnational data sharing and agreements to prioritize TB prevention and treatments. Better information and global cooperation alone are insufficient to curb TB impacts across populations. Countries must be willing to effectuate their own internal legal changes to address injustices.^22^ Among the key findings encapsulated in WHO’s Global Tuberculosis Report 2024 is the need to “[i]ntensify national efforts to create enabling *legal* and social policy frameworks to combat inequalities, and to eliminate all forms of TB-related stigma, discrimination and other human rights barriers and violations (emphasis added).”^23^

Although John Green may not focus on how modern legal reforms are key to TB prevention and control as other interventions, his text is not inherently flawed by this oversight. It’s just incomplete. Like a doctor telling how you need specific doses of a certain TB drug while failing to mention that the drug is only available through legal approval processes designed to assure its safety and efficacy. Or that the drug is not available at all due to legal impediments to access extending from health system or insurance limitations. Fixating too much on scientists, doctors, nurses, and philanthropists as premier sources of a “cure” looks past the integral roles of law- and policy-makers in preventing TB infections and resulting morbidity and mortality. “Everything is TB,” it seems, including the law.
